# Supplementary figures and images for: Characterizing changes in soil microbiome abundance and diversity due to different cover crop techniques
Source: PLoS One. 2020 May 5;15(5):e0232453. doi: 10.1371/journal.pone.0232453 (PMC7199946; doi:10.1371/journal.pone.0232453)

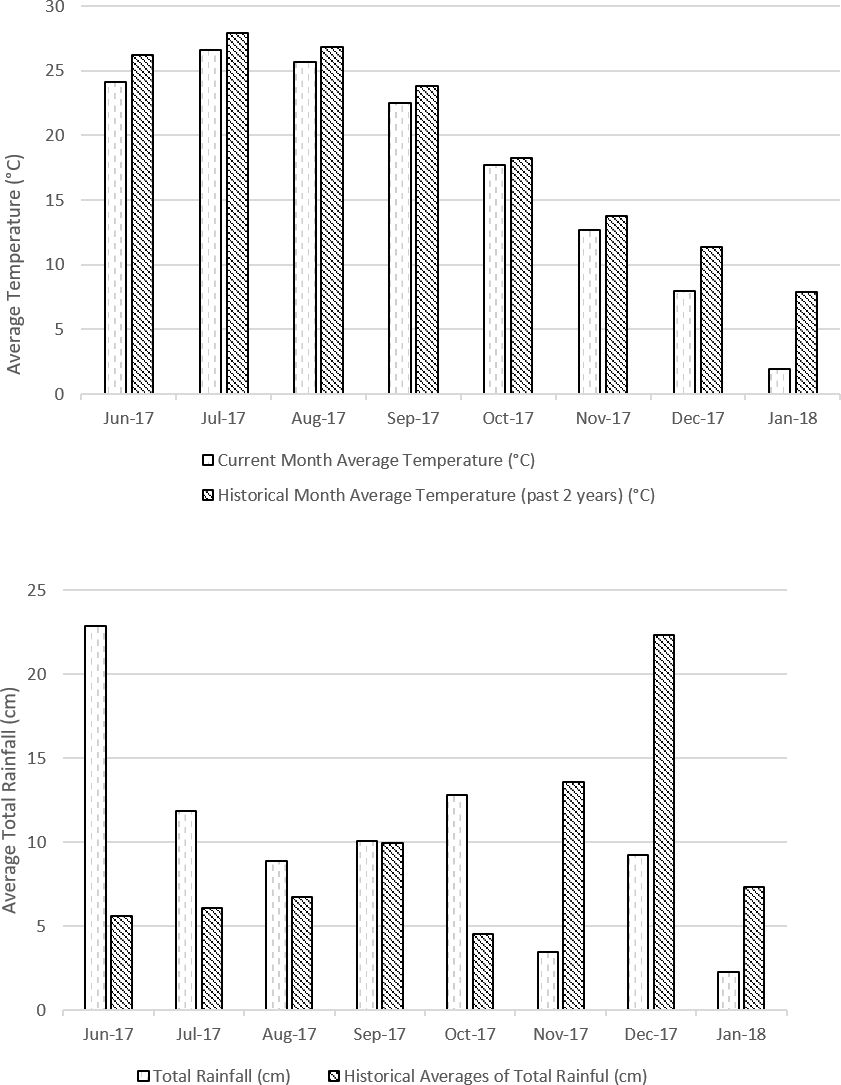

Supplement: S1 Fig — Average monthly temperature (A) and average total rainfall (B) in comparison to data from past two years. A decrease in average temperature was observed. The summer of 2017 had an increase in total rainfall, but the winter of 2017 was drier. (TIF) [file pone.0232453.s001.tif]

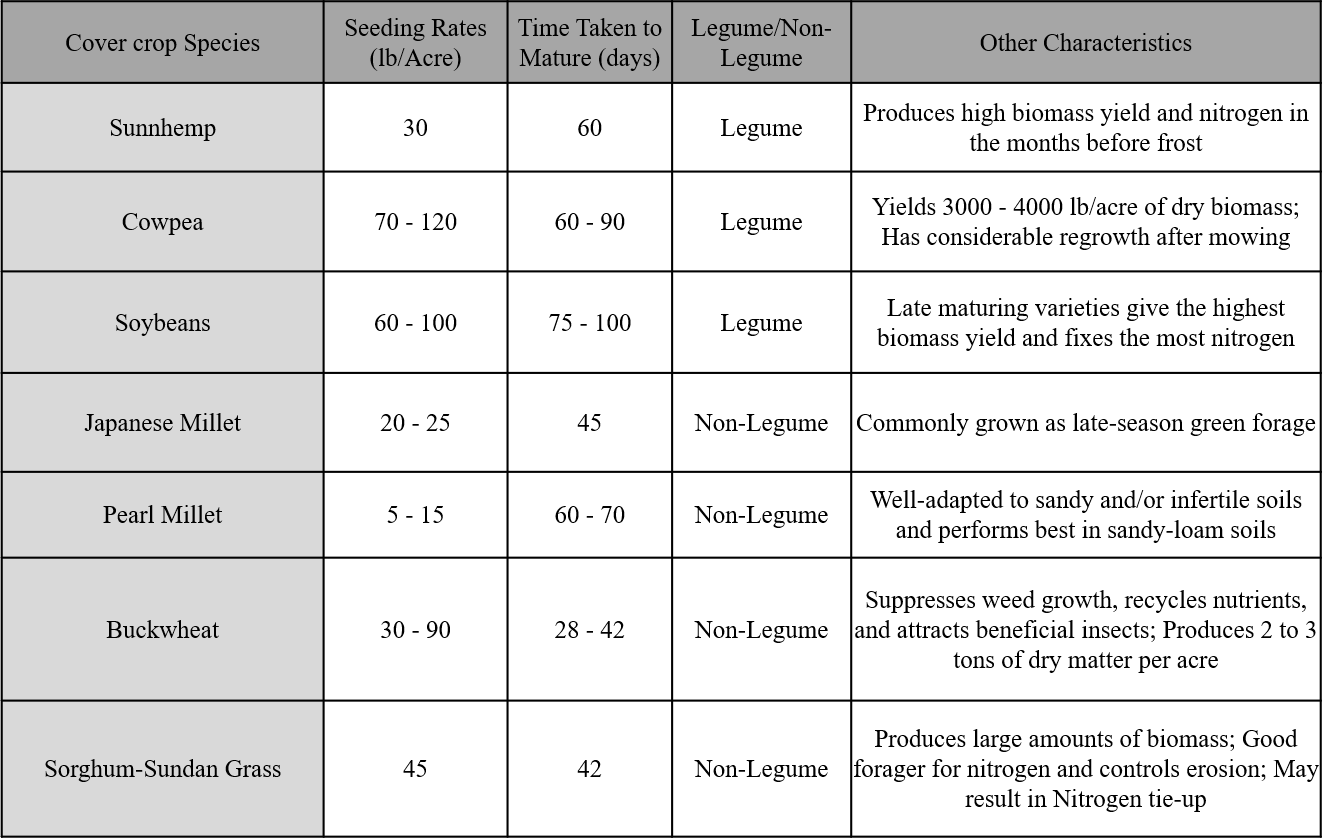

Supplement: S1 Table — (TIF) [file pone.0232453.s002.tif]

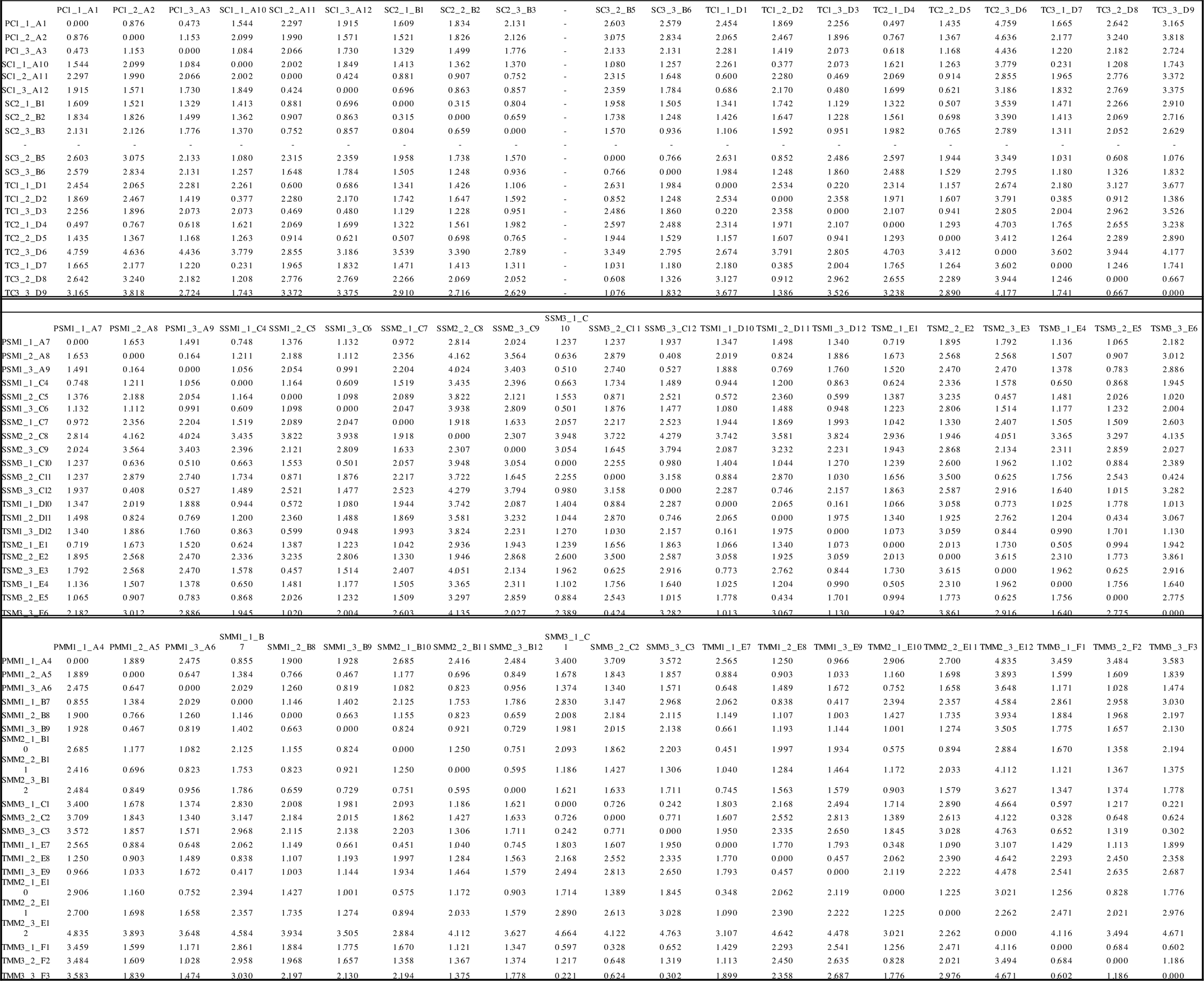

Supplement: S2 Table — (TIF) [file pone.0232453.s003.tif]

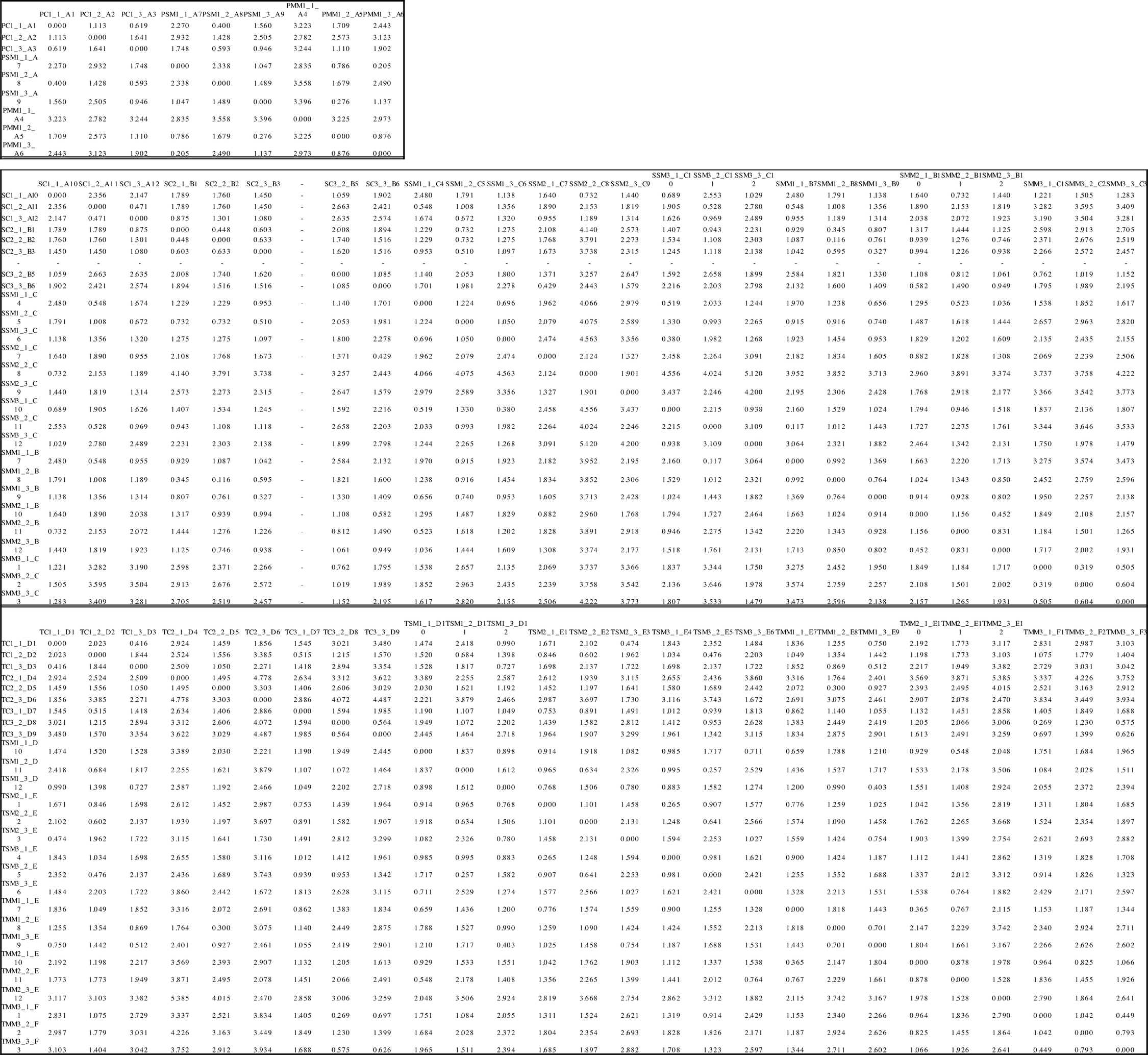

Supplement: S3 Table — (TIF) [file pone.0232453.s004.tif]
