# Supplementary material for: Characterizing changes in soil microbiome abundance and diversity due to different cover crop techniques
Source: PLoS One. 2020 May 5;15(5):e0232453. doi: 10.1371/journal.pone.0232453 (PMC7199946; doi:10.1371/journal.pone.0232453)
Supplement: Attachment — Submitted filename: Response to reviewers.pdf [file pone.0232453.s012.pdf]

EMORY
OXFORD COLLEGE

Sarah C. Fankhauser, Ph.D.
Assistant Professor of Biology

Division of Natural Sciences and Mathematics

Dear Dr. Proietti,

We are pleased to submit our revised manuscript entitled: *Characterizing changes in soil microbiome abundance and diversity due to different cover crop techniques.* We found the reviewer comments constructive and overall their comments have improved our paper. In addition to a revised manuscript, we have addressed each point below.

Sincerely,

Sarah Fankhauser, Ph.D.

Please ensure that your manuscript meets PLOS ONE's style requirements, including those for file naming.

We have reviewed the style requirements and ensured that files are named appropriately.

Please include captions for your Supporting Information files at the end of your manuscript, and update any in-text citations to match accordingly. Please see our Supporting Information guidelines for more information: http://journals.plos.org/plosone/s/supporting-information.

We have included captions for all supporting information files at the end of the manuscript as requested.

Have the authors made all data underlying the findings in their manuscript fully available?

The PLOS Data policy requires authors to make all data underlying the findings described in their manuscript fully available without restriction, with rare exception (please refer to the Data Availability Statement in the manuscript PDF file). The data should be provided as part of the manuscript or its supporting information, or deposited to a public repository. For example, in addition to summary statistics, the data points behind means, medians and variance measures should be available. If there are restrictions on publicly sharing data—e.g. participant privacy or use of data from a third party—those must be specified.

We are in the process of uploading our FASTA files to the Sequence Read Archive in Genbank

Reviewer #1: Dear Editor, the paper "Characterizing changes in soil microbiome abundance and diversity due to different cover crop techniques" is a study of the microbial composition of soils treated with single and multi-mix covercropping throughout a one year activity in organic agriculture regimen.

The article structure is sound, being organized around the hypothesis that multi-mix cover crop has an advantage on simpler cover crop choices. The statistics and the microbial methodology are correct. There are some major and minor points to improve, as listed below.

Major points

1. I have some doubts on the meaning and effect of these tiny experimental plots and if (even in the center of each plot) the border effect can cause problems of any type. In any case a discussion on this point seems necessary as well as the position of the sampling within the plot

We appreciate this reviewer's comments. We address these points in the methods and discussion sections and included additional references regarding the border effect (lines 803-813).

2. The OUTs are described only by stating that the standard QIME setting was used. In my experience, sometimes, QIME is indeed set up to define ASV rather than OTUs. The great number of OTUs makes me think that indeed they could be ASV groups. In any case, for clarity sake, the authors should state what was the similarity threshold chosen.

QIIME was set up to produce OTUs not ASV. The similarity cutoff in QIIME OTU is 97%. This has been addressed in the Methods section, line 249.

3. As an addition, I would also encourage the authors to briefly describe the variability within the OTUs in order to understand whether they are largely clonal or real OTUs with a species like structure.

Based on the similarity cutoff, OTUS that share ≥ 97% sequence similarity are collapsed into one, and this has been clarified in the methods (line 249). Given that the 16S genes are highly conserved, it is not a surprise that there is a high similarity level among the OTUs. We are concerned that more stringency in the OTU picking could over cluster and lead to a hiding of legitimate variations. Since our analysis is more focused on the higher taxonomical levels (like phyla), we don't believe we need to be so stringent at the OTU picking level.

4. The authors claim that the changes of the mixes are induced by variations in the rhizosphere, which is quite likely. Now the point is why did not the authors sample the soil among the roots separately from the rest if they wanted to propose and support this hypothesis? Is there any other indirect evidence in favor of this very likely hypothesis, beyond the likelihood itself?

Our samples were taken from the rhizosphere when appropriate, however time point one occurred before any planting and thus no rhizosphere was present. We have clarified our sampling technique in the methods section (lines 163-184).

5. The authors state at the beginning that this is one of the few papers on the one-year effect of cover cropping but fail to draw any conclusion about it in the course of the paper. For instance, the relative insensitivity or carbon content to the treatments, can be an effect of the short experimental time?

We appreciate this critique and have addressed this in the discussion and included relevant references and more discussion on the meaning of our results in terms of the experimental timeline (lines 578-592).

6. In general the paper should be a bit more condensed to ease its reading. The discussion is partly an enlargement of the result section, rather than a real discussion.

We have attempted to shorten and streamline the discussion to avoid too much of a reiteration of the results.

Minor points

1. Line 165- it is unclear how can the authors sample 500 ul, i.e half cubic centimeter, from a sampling cylinder with a 16 mm diameter and 100 mm depth. I would conclude that it is 64*pi *100 = 20.096 cubic millimeters i.e. ca 20 mL. In any case the author are encouraged to use Standard measures and therefore mm for lengths an mL or uL (no ul) for volumes.

We have changed the measurements in the methods as suggested by the reviewer (lines 184-187).

2. Line 199 – Fragment Analyzer. Is it the Agilent tool? If so state it, please, along with the experimental settings.

We used Fragment Analyzer System (Agilent) to assess the libraries size using the high sensitivity NGS kit. We have included this additional information in the methods (line 229).

3. Lines 208-209 – rephrase: it is hard to understand

This has been rephrased to improve clarity (now lines 244-246)

4. Line 218 – is it CRAN R anosim function? If so state it and acknowledge it with a proper citation. Moreover state its settings.

Statistical significance analysis: The "compare_categories.py" function in QIIME was used to analyze the strength and significance of the differences among samples of each group. We use the statistical method ANOSIM, which uses "bray" distance as the default dissimilarity function. The used dissimilarity function is "bray" [distance = bray]. This is the default function in the ANSIOM R function. We have updated our methods section to reflect these additions (lines 256-275).

5. ANOVA and KW were carried out with or what?

These statistical tests were carried out with our compositional relative abundance data. A more detailed explanation can be found below in response to Reviewer 2 #5.

6. Table 1, please separate thousands with the standard "," in order to make the figures more readable.

Table 1 has been modified with the standard ",". Page 12 line 350

7. Line 564 – sentence without the verb, apparently.

This has been resolved (currently now line 702)

8. Iconography taxonomic names not in italic.

This has been resolved.

Reviewer #2: Review

1. I don't believe the authors have submitted raw sequencing data to a public repository, such as NCBI SRA. While it might not be a strict requirement, doing so greatly improves the

reproducibility of the study and enables other researchers to improve on the analyses carried out by the authors.

We appreciate this reviewer's dedication to open access data. We are currently working on uploading the numerous FASTA files to SRA.

2. (Lines 162-163) The authors should explain, why there is only one replicate per subplot for the first time-point. Considering that the authors have used ANOVA and ANOSIM, both of which are quite sensitive to sample imbalances, the authors should emphasise this issue.

We have clarified this reasoning in the methods section. Ultimately, we chose 9 samples (one for each subplot) at the first timepoint since this was prior to any soil manipulation and thus we did not believe that more samples were necessary or cost effective.

3. (Lines 210-213) OTU picking is an extremely noisy process that is extremely prone to overestimating absolute diversity (i.e. the number of observed OTUs) [1,2,3,4]. Moreover, UCLUST is one of the least accurate heuristic sequence clustering algorithms [3]. The authors must apply denoising to reconstruct exact amplicon sequence variations using DADA2 [4] or Deblur.

The reviewer is right to be concerned about this noise in the OTU-picking process. While the ESV approach is much more stringent and uses only identical and unique 16S sequences in the downstream analysis, it has been shown to lead to 1) losing significant portion of the sequencing data due to its significance to data quality, 2) too much diversity, and hides/conceals the divergence in rRNA operons. This topic is discussed in details here (http://fiererlab.org/2017/05/02/lumping-versus-splitting-is-it-time-for-microbial-ecologists-to-abandon-otus/)

We did a comparison between DADA2 (ESV) and QIIME(OTU) on a portion of our data. Our goal was to assess if using either of the two methods leads to the identification of more/less/different phyla, given that our key results are at the phyla level. Our results are below and demonstrate that both approaches identify the same types and numbers of the top abundant phyla. Thus, we believe that the use of QIIME is appropriate for our data. ***We would be happy to include any of this as supplemental information, as deemed appropriate by the editor:***

**Timepoint1**	**QIIME**	**DADA2**
**Total # of references (OTU in QIIME and ESV from dada2)**	50,827	14,272
**Total # of unique phylum**	44 (a super set of data2's taxa)	26 (a subset of QIIME's taxa)
**Abundance comparison**	See Fig. 1, which is the Fig. 5a from the paper	See Fig. 2 (Taxonomic composition of the samples) and Fig. 3 log-ratio difference among groups

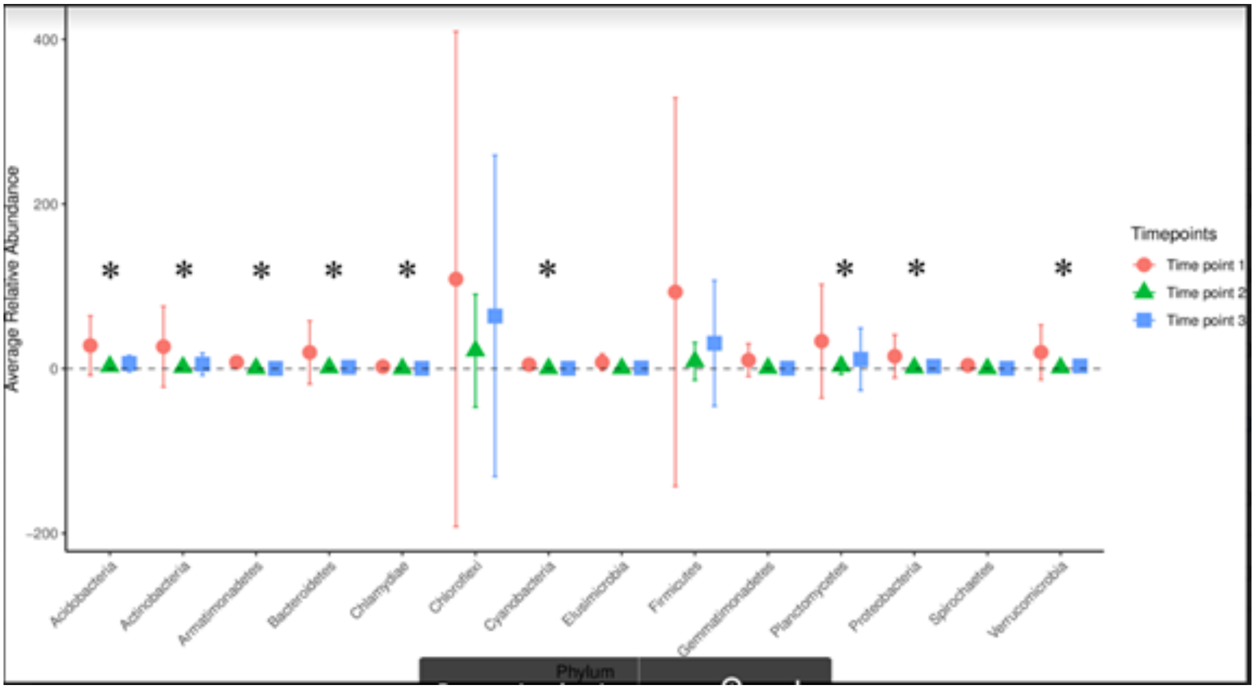

Fig. 1: This is Fig5a from the paper

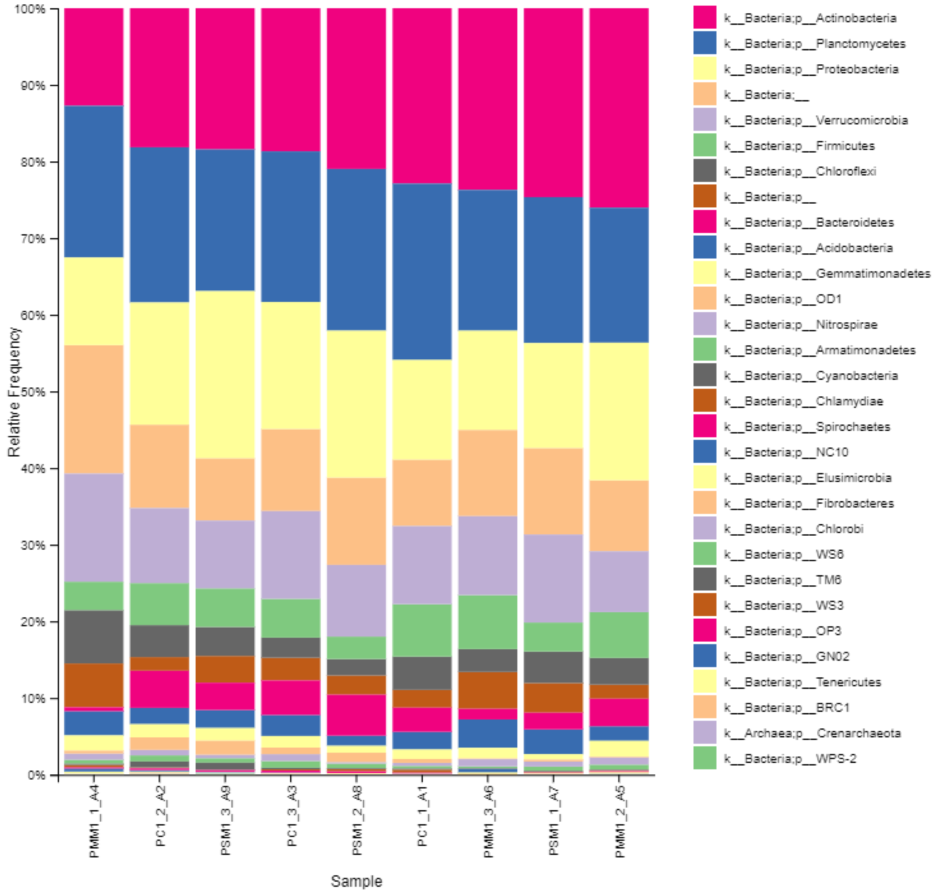

Fig. 2: Taxonomic composition of the samples in Timepoint1

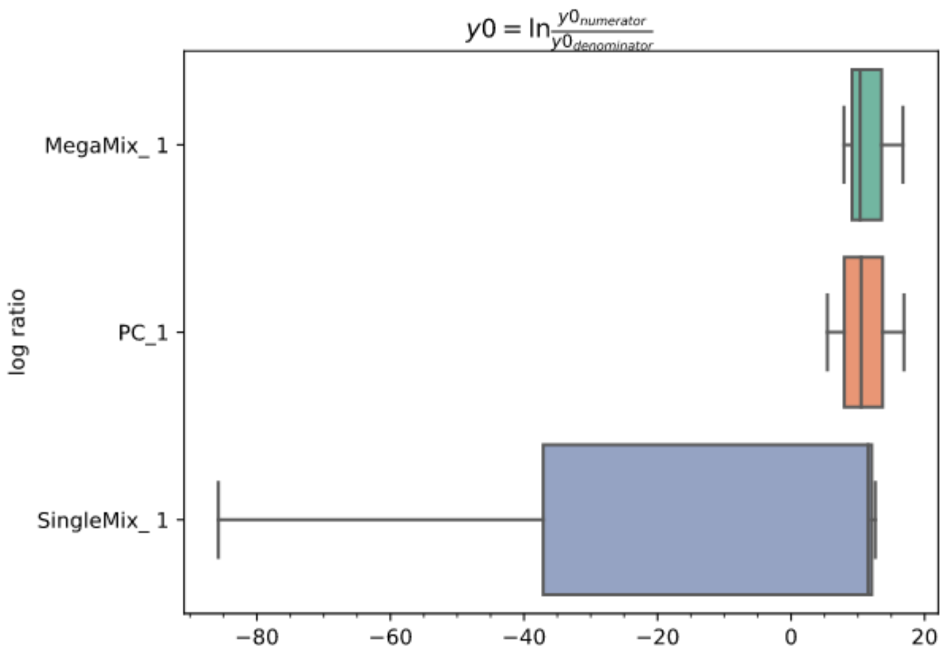

Fig. 3: A boxplot of log ratio of taxonomy in each treatment group. The ratio in each group is close but the Single Mixture group has more variety. The following plot is the taxonomy and its abundance in the numerator and denominator.

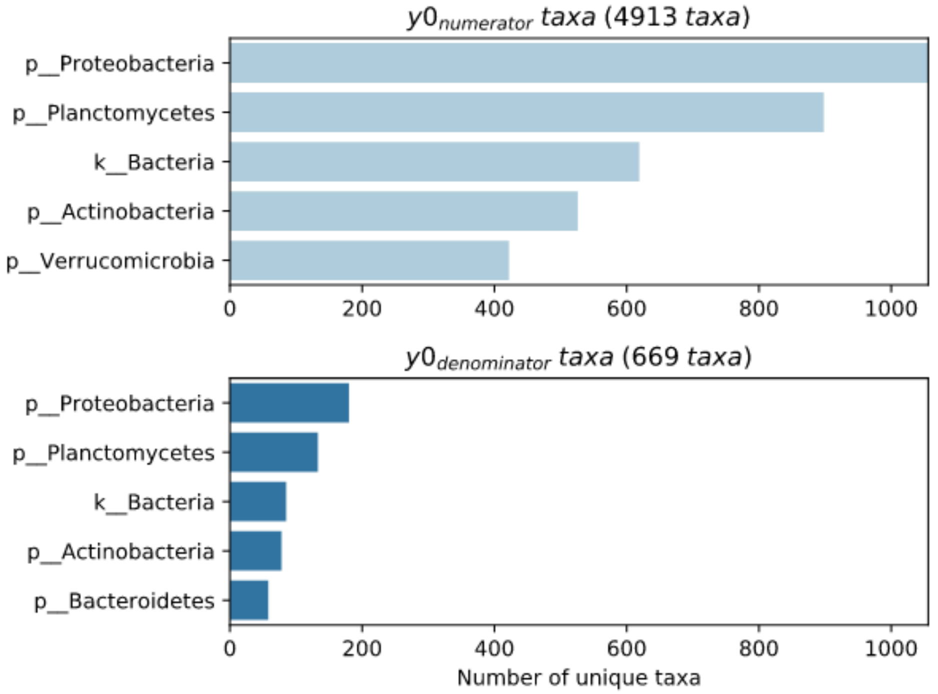

Timepoint2:

	QIIME	DADA2
**Total # of references (OTU in QIIME and ASV from dada2)**	79669	6745

**Total # of unique phylum**	51 (qiime is a super set of dada2)	31
**Abundance comparison**	See Fig. 1 - timepoint1	See Fig. 3 Taxonomic composition of the samples)

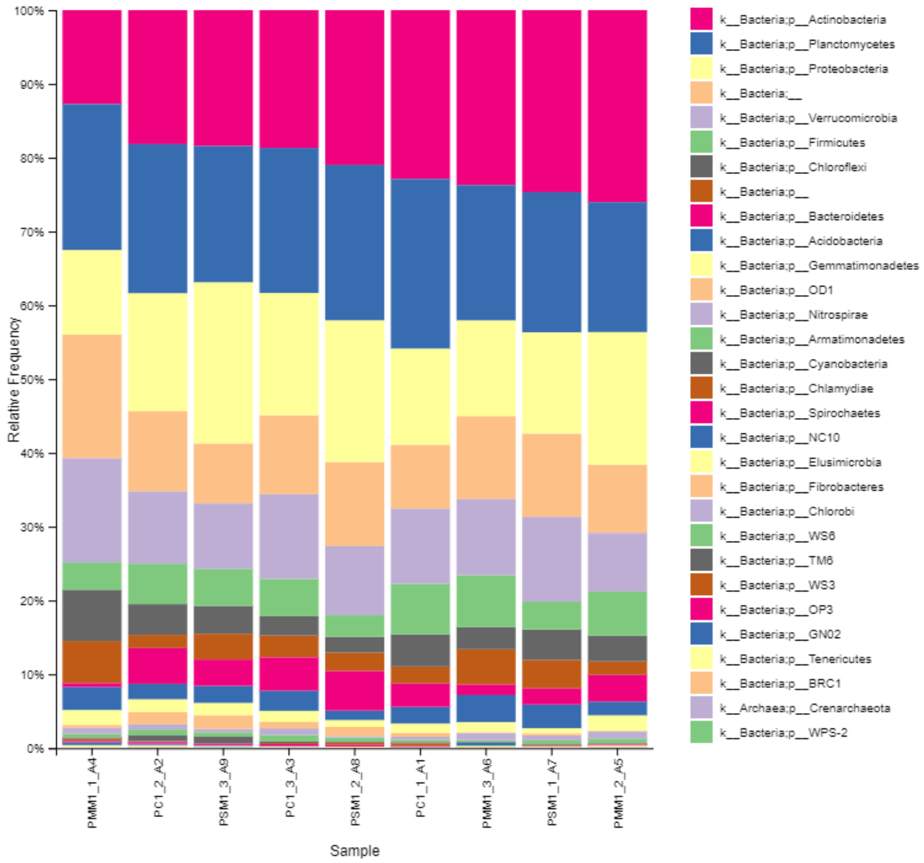

Fig.4: Taxonomic composition of the samples in Timepoint2

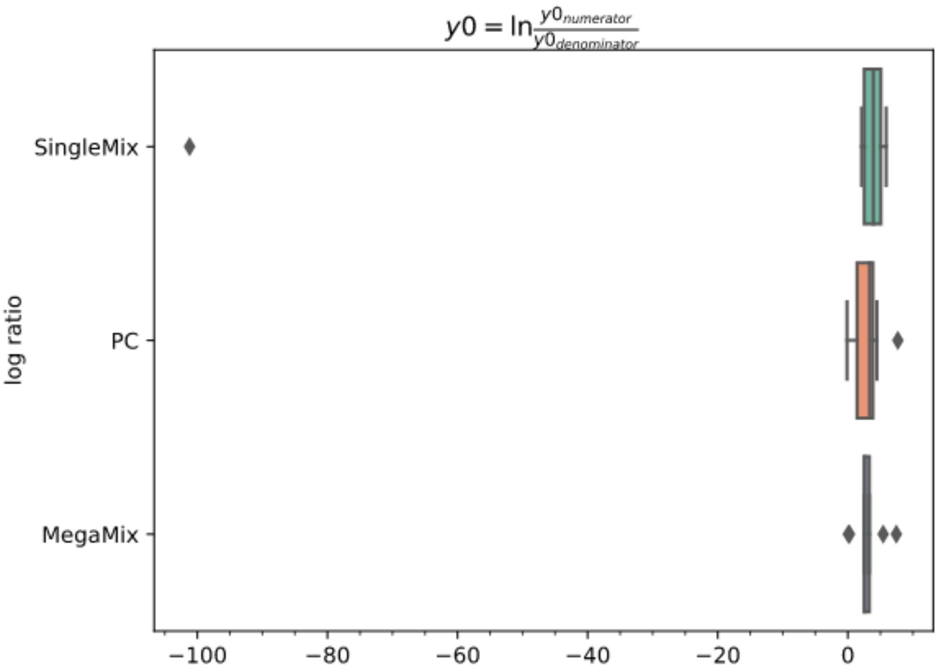

Fig. 4: A boxplot of log ratio of taxonomy in each treatment group. There no significant difference in Treatments. The following plot is the taxonomy and its abundance in the numerator and denominator.

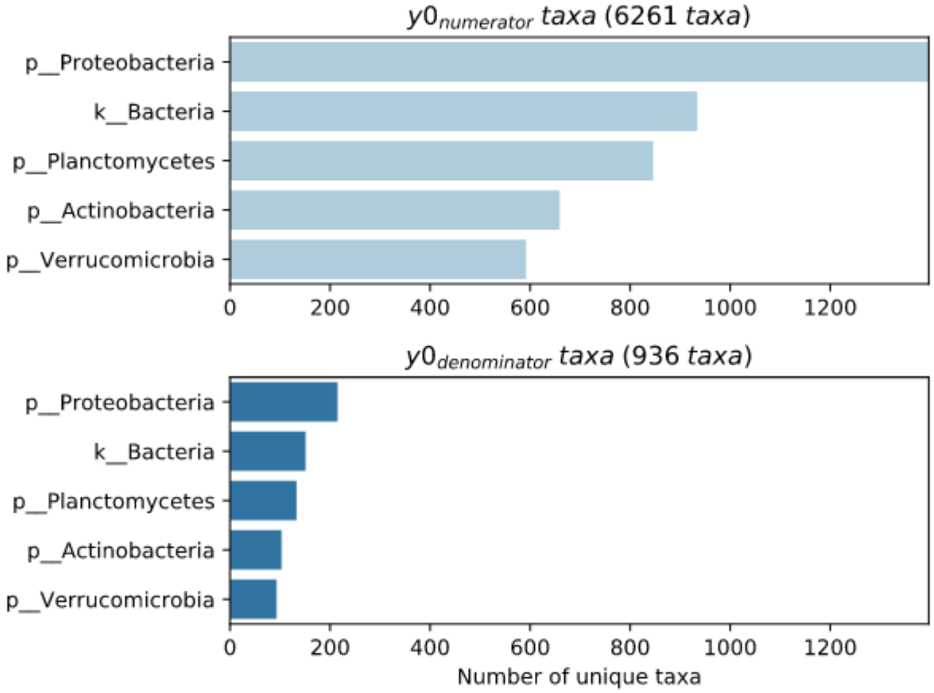

From the above comparative analysis, we conclude that top abundant phyla are the same in both methods, despite the differences in the level of abundance. In other words, the trend is the same in both analyses.

4. (Line 218) ANOSIM operates on dissimilarity matrices: the authors must specify the (dis)similarity functions they've used to generate these matrices.

The used dissimilarity function is "bray" [distance = bray]. This is the default function in the ANSIOM R function. This has been updated in more detail in the methods section (lines 256-275).

5. (Lines 219-221, 229) ANOVA, Kruskal-Wallis and t-test cannot be applied to raw compositional data, such as sequencing data and chemical compositions [5,6]: the authors must carry out statistical analyses designed for compositional data.

We appreciate this critique and the references provided. We would like to refer the reviewer to the following two resources:

1. *Hypothesis testing and statistical analysis of microbiome: https://www.ncbi.nlm.nih.gov/pmc/articles/PMC6128532/*
2. *QIIME: http://qiime.org/scripts/group_significance.html*

The above-mentioned statistical methods and their suitability for the microbiome sequencing data are discussed. In addition, these methods are applied on normalized count matrix, which is the same as RNAseq and microarray data at this point. These statistical methods are routinely used for significance analysis between groups, and as such we believe that this is the appropriate statistical approaches for our dataset. We hesitate to add these explanations to our methods section given the present length of the manuscript, however if the editor thinks this would be a valuable addition then we would be happy to add it.

6. (Lines 271, 283-285) Absolute diversity analysis is neither reliable (due to aforementioned issues with OTU picking), not subcompositionally coherent. The same critique applies to Chao and Shannon indices. If the authors do want to conduct alpha-diversity analysis, they can use the Aitchison's norm. Otherwise, alpha-diversity analyses must be removed.

To estimate both alpha and beta diversity, we used the QIIME functions alpha_rarefraction.py and beta_diversty.py, respectively. These functions do normalize the data using the rarefaction method. This is described in detail here:

1. *Alpha diversity: http://qiime.org/scripts/alpha_diversity.html*
2. *Alpha diversity analysis: https://twbattaglia.gitbooks.io/introduction-to-qiime/content/alpha_analysis.html*
3. *Beta diversity: http://qiime.org/scripts/beta_diversity.html*

We made a quick comparison between Rarefaction and Aithchison normalization methods in two time points, see the following tables and plots:

Timepoint1 Anosim

	**Rarefaction**	**Aitchison**
test statistic	0.061728395061728371	0.152263
p-value	0.038	0.162

Timepoint2 Anosim

	Rarefaction	Aitchison
test statistic	0.0618729	0.000248016
p-value	0.154	0.425

Overview

	ANOSIM results
method name	ANOSIM
test statistic name	R
sample size	25
number of groups	3
test statistic	0.0618729
p-value	0.116
number of permutations	999

Group significance plots

Download raw data as TSV (raw_data.tsv)

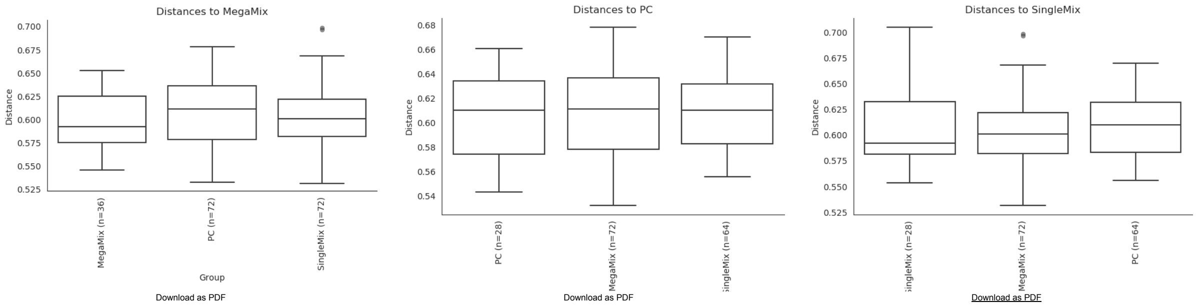

Download as PDF (MegaMix-boxplots.pdf) | Download as PDF (PC-boxplots.pdf) | Download as PDF (SingleMix-boxplots.pdf)

Overview

	ANOSIM results
method name	ANOSIM
test statistic name	R
sample size	24
number of groups	3
test statistic	0.000248016
p-value	0.425
number of permutations	999

Group significance plots

Download raw data as TSV (raw_data.tsv)

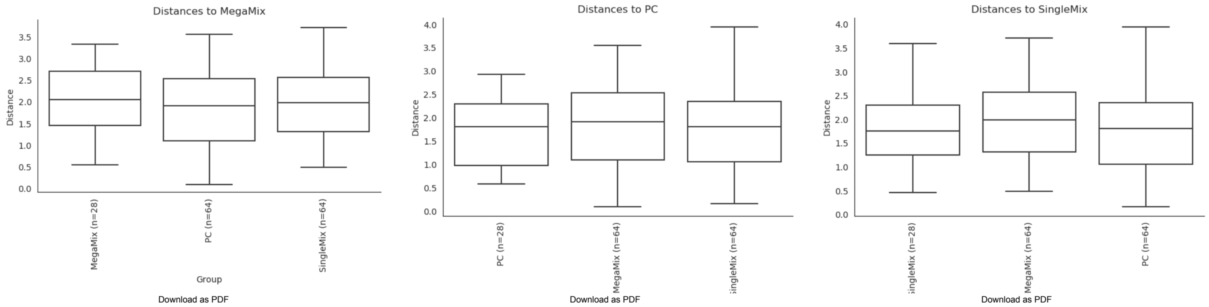

Download as PDF (MegaMix-boxplots.pdf) | Download as PDF (PC-boxplots.pdf) | Download as PDF (SingleMix-boxplots.pdf)

In timepoint 1, the statistical tests show that both rarefaction and Aitchison are different. However, both methods produce similar statistical tests (in terms of significance) in timepoint 2. We therefor think that using the rarefaction normalization prior to alpha and beta diversity estimation is as good as the Aitchison normalization. ***If the editor deems it appropriate, we are glad to add these explanations to the methods section of the paper or as supplemental data.***
