# Supplementary material for: Characterizing changes in soil microbiome abundance and diversity due to different cover crop techniques
Source: PLoS One. 2020 May 5;15(5):e0232453. doi: 10.1371/journal.pone.0232453 (PMC7199946; doi:10.1371/journal.pone.0232453)
Supplement: Attachment — Submitted filename: PLoS response to reviewers.docx [file pone.0232453.s013.docx]

Dear Dr. Proietti,

We are pleased to submit our revised manuscript entitled: *Characterizing changes in soil microbiome abundance and diversity due to different cover crop techniques.* We found the reviewers’ persistence and feedback ultimately very constructive and you will see that we have made substantial changes to our methodology and analysis, as recommended by reviewer two. In addition to a revised manuscript, we have addressed each point below.

-----------------------------------------------------------------------------------------------------------------

Reviewer #1: The authors amended quite well their paper and gave exhaustive and satisfactory answers to the question posed.

Reviewer #2: 1. We appreciate this reviewer’s dedication to open access data. We are currently working on uploading the numerous FASTA files to SRA.

Thank you very much for this decision. I am looking forward to seeing your data uploaded to SRA.

*Given the reanalysis of our data, and the new files, we are still in the process of uploading our fastq data. We have also included substantial amount of supplemental data in our submission.*

2. I believe, you refer to the following sentence (lines 159-161): "There was only 1 sample per subplot taken at time point 1 since the soil had just been evenly tilled across each subplot and no further manipulation had been undertaken; thus the extra replicates of the plots were deemed unnecessary".

Even if that's the case, you should have considered the effect sample imbalance has on statistical methods you've chosen. ANOSIM, in particular, is extremely sensitive to sample size imbalance and heteroscedasticity (that accompanies sample imbalances) [1]. If you are going to keep ANOSIM in your analysis, you should leave an appropriate warning about sample imbalances.

*We have noted the limitation of this analysis in the methods, lines 160-163.*

3. I have given multiple peer-reviewed references showing how bad OTU-picking, (and UCLUST-based OTU-picking in particular) is at recovering microbial diversity from amplicon sequences. Considering that you are working with the total number of OTUs in your analysis, the fact that OTU-picking has been shown to dramatically (from 10^1 to 10^3 times) inflate this number is absolutely damning, even if the procedure produces adequate composition at the level of phyla. With all due respect, a single unreviewed blog-post is not a valid source of evidence. Moreover, the very people behind QIIME's original preprocessing pipeline implore their users to abandon it in favour of denoising: "... This process, also known as OTU picking, was once a common procedure, used to simultaneously dereplicate but also perform a sort of quick-and-dirty denoising procedure (to capture stochastic sequencing and PCR errors, which should be rare and similar to more abundant centroid sequences). Use denoising methods instead if you can. Times have changed. Welcome to the future" [ https://docs.qiime2.org/2019.10/tutorials/overview/ ].

*We thank the reviewer in their persistence with this, and we have heeded the advice. We have completely reanalyzed the data using QIIME 2 and performed a denoising step (described in the methods lines 219-221 and in the results lines 287 and 301 ). This is further represented in Table 1. We believe that this reanalysis has revealed new and interesting trends which we discuss extensively in our results and discussion sections.*

4. Bray-Curtis is not subcompositionally coherent – you should add an appropriate warning. You might consider adding another ANOSIM assay based on Aitchison distances.

*We have performed an ANOSIM on the Aitchison distances, the Aitchison distance values are included in supplemental tables 2 and 3 (S2 Table, S3 Table), and the resulting processed data was analyzed using multivariate comparative methods such as ANOVA and ANOSIM, the results were reported in results Tables 2A and 2B.*

5. I am not sure you have paid due attention to my references, but I will return to them later. As for the references you have provided, the second one is a link to QIIME1 documentation. Since QIIME1 is a woefully outdated piece of software that has been discouraged by its own authors, its documentation doesn't look like a valid reference to rely on. That leaves the first reference, i.e. "Hypothesis testing and statistical analysis of microbiome" by Xia and Sun. First of all, this paper is a review of methods that have been used in microbiome research regardless of statistical correctness. Second of all, the very paper contains the following sentences:

"First, the existing statistical methods for analyzing microbiome proportional data do not solve constraint problem, and some researchers even do not know it exists. Most standard statistical methods, such as the Pearson correlation coefficient, t-test, ANOVA are still widely used or exist in current literature on the analysis of microbiome data[23, 24, 25, 26] without testing the data distribution and transformation. One assumption of standard statistical methods is that the compared data are independent. Since the sum of the relative abundances is unity, it indicates the data are not independent with the unity or any constant constrain. Thus it is not appropriate to directly use these methods for analyzing microbiome relative abundance data".

In other words, your own reference reinforces my line of criticism. The fact that you are using a "normalized count matrix, which is the same as RNAseq" changes nothing, because no normalisation procedure can remove the total-sum-constraint problem (it can only change the total sum). Furthermore, RNAseq methods operate under strong assumptions (i.e. there must be many transcripts, but few of them can vary across states) that simply do not apply to microbiome data (whether these assumptions hold in RNAseq studies is a separate issue). Even ALDEx2 is not ideal for microbiome research, because it simply applies the CLR transform (which is similar to the normalisation procedure from DESeq2) and operates under the same assumptions as other RNAseq packages. This is, of course, covered in depth in the references I have given in the previous response.

Here I would like to reiterate a line from my previous response: the authors must carry out statistical analyses designed for compositional data.

*We have conduced statistical analyses designed for compositional data by transforming our data to Aitchison’s distances. We used the ANOSIM and ANOVA method to test whether the treatment among 3 groups are significantly different. According to the reviewer 2’s suggestion, the Aitchison’s distance is applied to calculate the distance matrix (the documentation is here: https://library.qiime2.org/plugins/deicode/19/ ). This is described in both the methods (starting on line 224) and results sections (starting on line 319).*

6. It is a norm, not a normalisation procedure. How have you treated zeros?

*We have described our analysis, using Aitchison norm, starting on line 224 (statistical anlaysis) in the methods. We provide additional details on our use of Aitchison norm in the results section. We used the cutoff at 1 for each feature, so it will ignore the features with 0 count.*
